# Functional Analysis of Complex Structural and Splice-Altering Variants in the *ARSB* Gene Towards the Personalized Antisense-Based Therapy for Mucopolysaccharidosis Type VI Patients

**DOI:** 10.1155/humu/2250030

**Published:** 2025-01-10

**Authors:** Igor Bychkov, Alexandra Filatova, Galina Baydakova, Nataliya Sikora, Emiliya Garifullina, Anna Bykova, Vyacheslav Tabakov, Alexandr Skretnev, Mikhail Skoblov, Ekaterina Zakharova

**Affiliations:** ^1^Department of Molecular Mechanisms of Inherited Metabolic Disorders, Research Centre for Medical Genetics, Moscow, Russia; ^2^Department of Functional Genomics, Research Centre for Medical Genetics, Moscow, Russia; ^3^Department of Medical Genetic Consultation, Perinatal Center Named After Professor G.S. Postol, Khabarovsk, Russia; ^4^Department of Medical Genetic Consultation, Republican Medical Genetics Center, Ufa, Russia; ^5^Department of Medical Genetic Consultation, Arkhangelsk Regional Children's Clinical Hospital Named After P.G. Vyzhletsov, Arkhangelsk, Russia; ^6^Children's Psychoneurological Department, Children's Regional Clinical Hospital Named After A.K. Piotrovich, Khabarovsk, Russia

**Keywords:** antisense therapy, DNA recombination, Maroteaux–Lamy syndrome, next-generation sequencing, premature polyadenylation, pseudoexon, splicing correction

## Abstract

Mucopolysaccharidosis Type VI (MPS VI) is a lysosomal storage disorder associated with biallelic pathogenic variants in the *ARSB* gene. Herein, we present three patients with biochemical and clinical pictures of MPS VI, for whom routine molecular genetic analysis using Sanger sequencing of *ARSB* failed to identify one or both causative variants. RNA analysis of patients' samples revealed alterations of the wild-type *ARSB* mRNA isoform in all cases, and one case required further analysis using whole genome sequencing. As a result, we identified one complex structural variant, which is a 52-kb insertion of the *LHFPL2* gene fragment in the *ARSB* Intron 4, derived from nonallelic homologous recombination and leading to premature transcription termination, a recurrent deep intronic variant leading to pseudoexon activation and an intragenic deletion altering the integrity and splicing of the *ARSB* Exon 2.

Using a minigene-based cellular model, we demonstrated that the identified pseudoexon can be efficiently blocked by antisense molecules incorporated into modified U7 small nuclear RNAs and circular RNAs. The same approach was used to block the overlapping polymorphic pseudoexon in the *ARSB* gene and increase the amount of wild-type mRNA isoform approximately twofold.

## 1. Introduction

Mucopolysaccharidosis Type VI (MPS VI) or Maroteaux–Lamy syndrome (MIM: 253200) is a rare autosomal recessive disease from the group of lysosomal storage disorders. MPS VI is caused by a deficiency of the lysosomal enzyme aryl-sulfatase B (ASB), encoded by the *ARSB* gene (NM_000046.5). ASB is involved in the degradation of glycosaminoglycans (GAGs)—dermatan sulfate and chondroitin-4-sulfate. Accumulation of these GAGs in the cells' lysosomes and extracellular matrix leads to progressive damage to cells and tissues with subsequent development of multisystemic disorder. Clinical phenotypes of patients with MPS VI represent a continuum, ranging from severe early-onset forms with rapid progression of osteoarticular signs to later-onset slowly progressing forms, which are often noted only by the presence of coarsened facial features [1].

The diagnosis of MPS VI is primarily based on quantitative analysis of urinary GAGs, detection of the reduced activity of ASB in blood samples, and Sanger sequencing of eight exons of the *ARSB* gene. The published study analyzing 478 individuals diagnosed with MPS VI demonstrated that the *ARSB* mutational spectrum mainly comprises missense variants (59.5%), nonsense variants (12.0%), and small deletions (13.5%) [2]. The structural variants, which are difficult to detect by routine molecular-genetic analysis, were presented only in three cases, and in 9.2% of patients, only one pathogenic allele has been reported. Several studies implemented the *ARSB* mRNA analysis to increase the molecular genetic diagnostics yield, which led to the identification of deep intronic pathogenic variants or reduced *ARSB* expression [3, 4]. The importance of timely and accurate diagnosis of MPS VI is emphasized by the presence of enzyme replacement therapy and hematopoietic stem cell therapy [5, 6].

In this article, we demonstrate the application of RNA analysis and a number of molecular genetic techniques that may assist researchers in establishing the definitive genetic diagnosis of MPS VI in cases where Sanger sequencing failed to identify causative variants in the *ARSB* gene. RNA analysis has provided us with clues to detect and functionally characterize such rare types of disease-causing variants as large insertions derived from nonallelic homologous recombination (NAHR), intragenic deletion, and pseudoexon (PE)-activating deep intronic variant.

Deep intronic variants, which alter genes' splicing by activating PEs, have recently become of particular interest. The field of antisense therapy has acquired a large number of effective instruments for correcting such types of mutations. In addition to conventional approaches based on the delivery of chemically modified antisense oligonucleotides, researchers have begun to actively employ antisense molecules incorporated into modified U7 small nuclear RNAs (modU7snRNAs) [7, 8] or circular RNAs (circRNAs) [9]. This approach allows to effectively deliver antisense molecules to a wide range of tissues by adeno-associated viral vectors, offering additional therapeutic options for MPS VI patients.

## 2. Results

### 2.1. Patient 1: Insertion of the *LHFPL2* Gene Fragment Derived From NAHR

Patient 1 is a 5-year-old female referred to a geneticist due to severe growth retardation, kyphoscoliosis, and contractures of the hands.

The patient was born at 36 weeks of gestation with a birth weight of 2560 g and length of 50 cm. Prolonged jaundice was observed in the first week of life. At 1 month of age, a patent arterial duct and a patent foramen ovale were discovered. Successful surgery for the arterial duct occlusion was performed at 2 months. At 1.5 years, the parents noticed contractures in the child's knee joints and growth retardation.

At the age of 5 years, clinical examination demonstrated failure to thrive (weight: 13.2 kg; standard deviation score (SDS): −2.7), marked growth retardation (height: 86 cm; SDS: −4.47), valgus deformities of the lower extremities, contractures at the knees, hips, and interphalangeal joints, as well as kyphoscoliosis and pectus carinatum. The patient exhibited distinctive facial features, including coarse facies, hypertelorism, exophthalmos, a depressed nasal bridge, a broad nose, thick lips, and a prominent forehead. Hepatosplenomegaly was also present (liver palpable 6 cm below the costal margin; spleen 3 cm below the costal margin). At 5 years, the patient had mild mental retardation, and her speech was limited to short sentences and simple words. Echocardiography revealed an early stage of the left ventricular hypertrophy, the mitral valve stenosis and prolapse, and aneurysm of the interatrial septum. Ophthalmologic examination showed bilateral mild corneal clouding, high hypermetropia, and hypermetropic astigmatism. Radiological examination showed multiple skeletal abnormalities (dysostosis multiplex), “J-shaped” conformation of the sella turcica, platyspondylia, and deformation of the vertebrae, along with the odontoid process hypoplasia. The epiphyses of the long bones were hypoplastic with cortical thinning and osteopenia, while the carpal bones were hypoplastic and irregularly shaped. Metacarpal bones were proximally tapered, shortened, and thickened. The results of biochemical tests revealed the reduced activity of ASB in dried blood spots (0.001 *μ*mol/L/h, normal range 1–15 *μ*mol/L/h) and an increase of dermatan sulfate in urine, which are biomarkers of MPS VI.

Sanger sequencing of the patient's *ARSB* gene identified three heterozygous variants: rare synonymous variant c.783G>A (p.Lys261=) in the Exon 4, pathogenic nonsense variant c.966G>A (p.Trp322⁣^∗^), and common missense variant c.1072G>A (p.Val358Met) in the Exon 5. Segregation analysis revealed that all three variants were inherited from the patient's mother.

As the rare synonymous c.783G>A (p.Lys261=) variant was not described earlier, its potential effect on splicing in the absence of nonsense-mediated decay (NMD) was analyzed by minigene assay (Note S1 and Figure S1). The results demonstrated that c.783G>A has no significant effect on splicing and therefore, considering its synonymous nature, was classified as “likely benign”.

**Figure 1:**
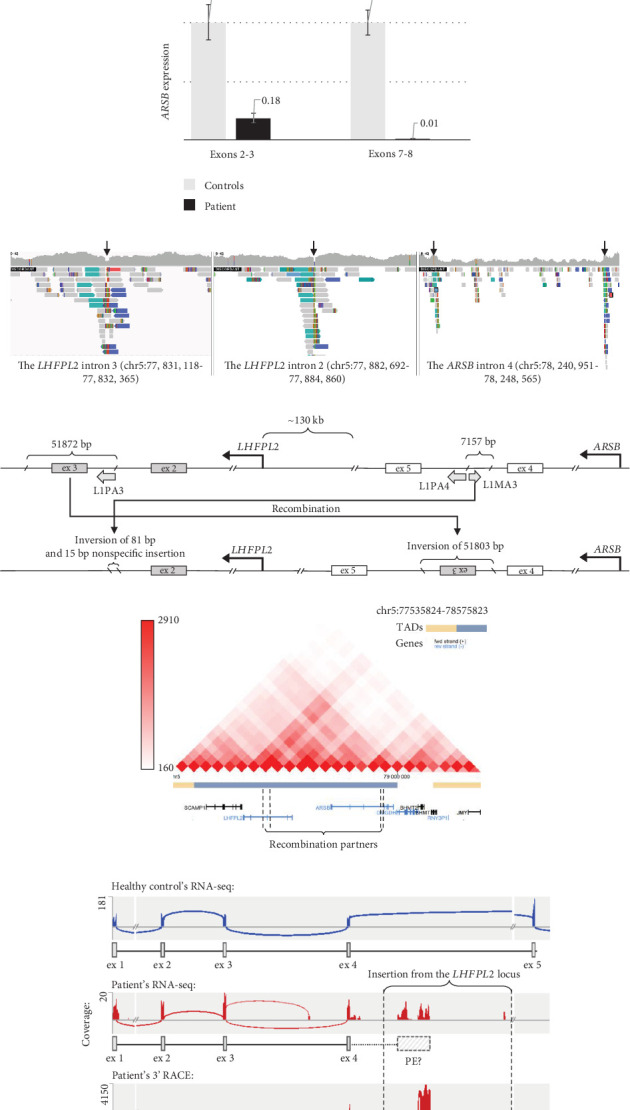
Molecular genetic findings in Patient 1. (a) Analysis of patient's cDNA sample by PCR and Sanger sequencing. Three heterozygous variants, presented in a patient in cis-position, were amplified with primers f1, r1 for the c.783G>A variant and f1, r2 for c.966G>A and c.1072G>A variants. Sanger chromatograms of a patient's *ARSB* cDNA fragments demonstrate the specific for PTT allelic imbalance. (b) The results of real-time PCR with primers spanning Exons 2–3 and 7–8 of *ARSB* (“control” bar represents the mean expression of *ARSB* in three healthy unrelated control samples). (c) The IGV browser window demonstrating groups of discordant reads in *LHFPL2* and *ARSB* and reduction of the sequence coverage between breakpoints in *ARSB*, identified during whole genome sequencing analysis. Breakpoints are indicated by arrows. (d) The scheme of DNA region at Chromosome 5 containing *LHFPL2* and *ARSB* genes before and after the recombination event. Gray arrows indicate mobile genetic elements and their orientation. (e) The Hi-C heat map of DNA interactions in the GM12878 cell line (http://3dgenome.fsm.northwestern.edu/) showing that recombination partners are located in the close spatial proximity. (f) Sashimi plots representing the results of RNA-seq and deep sequencing of 3⁣′ RACE products. A group of RNA-seq reads is mapped to the 5⁣′ end of the insertion, which suggests the presence of a PE with uncertain boundaries (indicated as “PE?”). 3⁣′ RACE determined the exact boundaries of the exonized sequences and revealed two PEs (indicated as “PE5 + PE6”) divided by the 132-bp intron.

To search for a pathogenic variant on the second chromosome, which can alter the gene's expression or splicing, the *ARSB* mRNA analysis was performed. Reverse transcription–polymerase chain reaction (RT-PCR) analysis with primers amplifying the whole *ARSB* cDNA by overlapping fragments did not reveal any additional mRNA isoforms, but the characteristic allelic imbalance was detected (Figure 1(a)). Sanger sequencing of *ARSB* cDNA fragments containing heterozygous variants demonstrated that peaks from the mother's allele are significantly lower before the Exons 4–5 junction and higher after it. Considering that the expression of the mother's allele was severely reduced by NMD, these results suggested that the second pathogenic allele represents a variant located in the intron 4, which leads to premature transcription termination (PTT). The results of quantitative polymerase chain reaction (qPCR) with primers spanning Exons 2–3 and 7–8 also supported this hypothesis, as it turned out that the expression of the full-length mRNA isoform was almost absent and the expression of isoforms containing Exons 2–3 was reduced to 18% (Figure 1(b)).

To identify the variant, which caused the PTT, whole genome sequencing (WGS) was performed. The structural variant analysis identified two DNA breakpoints in the *ARSB* intron 4 with groups of discordant reads, whose mates were mapped to the *LHFPL2* gene (Figure 1(c)). In addition, a decrease in the sequencing coverage between breakpoints in *ARSB* was observed. An unbalanced translocation was suspected and further confirmed by Sanger sequencing of breakpoints in both genes. During the translocation event, the 51,872 bp fragment (chr5:77,831,892–77,883,763) was removed from the *LHFPL2* gene, and 51803 bp of it (chr5:77,831,940–77,883,742) was inserted in the reverse-complement orientation in the *ARSB* Intron 4 (Figure 1(d)). In the *ARSB* gene, the 7157 bp fragment of the intron 4 (chr5:78,241,273–78,248,429) was removed and only 81 bp of it (chr5:78,248,357–78,248,437) in the reverse-complement orientation together with a nonspecific 15 bp sequence located downstream were detected in *LHFPL2* (Note S2).

Analysis of translocation breakpoints revealed two highly homologue regions, containing mobile genetic elements of L1PA3, L1PA4, and L1MA3 classes (Figure 1(d)). The identity between the L1PA3 element in *LHFPL2* and the L1PA4 element in *ARSB* reaches up to 93% over 5187 bp. The identity between the L1PA3 element in *LHFPL2* and the L1MA3 element in *ARSB* reaches 71% over 1614 bp. Furthermore, the regions involved in translocation, are located in the close spatial proximity to each other, being parts of the same topologically associated domain (Figure 1(e)). Both these facts are highly favorable for homologous recombination. Thus, the identified translocation can be further characterized as NAHR. Analysis of the DNA of patient's parents with breakpoint-specific primers revealed that the recombination occurred in the patient de novo.

To evaluate the effect of the identified recombination on the *ARSB* mRNA structure, we performed high-throughput sequencing of RNA (RNA-seq) extracted from cultured fibroblasts of the patient and healthy nonrelated control. We found that the total *ARSB* gene expression was reduced by 4.7 times in the patient's sample compared to the control (Table S1). Moreover, the patient's *ARSB* transcript was truncated and included only wild-type (WT) Exons 1–4 (Figure 1(f)), with r.783G allele comprising 88% of reads (Figure S2). These observations are consistent with the previously obtained data on the allelic imbalance and the *ARSB* expression measured by qPCR (Figures 1(a) and 1(b)) and support the hypothesis that the NAHR-derived insertion leads to PTT after the Exon 4. We also observed a group of RNA-seq reads aligned to the insertion, however, we could not accurately establish the 3⁣′ end of the chimeric transcript based on these data due to low expression and complex repeat architecture of the locus.

The RNA-seq data were also used to analyze the expression and the mRNA structure of the *LHFPL2* gene. We revealed that its total expression did not change in patient's fibroblasts relative to the control. However, the *LHFPL2* Exon 3 skipping was observed in approximately half of the transcripts, consistent with the heterozygous state of the deletion in *LHFPL2* encompassing this noncoding exon (Figure S3).

To precisely establish the 3⁣′ end of the chimeric *ARSB* transcript, rapid amplification of cDNA ends (RACE) was performed on the same RNA sample. Deep sequencing of obtained PCR products revealed that the *ARSB* Exon 4 was spliced with two PEs located near the 5⁣′ end of the *LHFPL2* insertion (Figure S4). The first 67 bp PE (corresponding to chr5:77834236-77,834,302) has both strong acceptor and donor splice sites and is spliced with a strong acceptor site of the second terminal ~715 bp PE located 132 bp downstream (corresponding to chr5:77834435-77835149). The strong polyadenylation (polyA) signal AATAAA is located at the 3⁣′ end of the terminal exon and 18 bp downstream the chimeric transcript ends with a polyA tail (Note S3).

### 2.2. Patient 2: Intragenic Deletion Altering the Integrity and Splicing of the *ARSB* Exon 2

Patient 2 is a male infant referred to a geneticist at the age of 2 years and 10 months with spinal deformities.

The boy was born from the third pregnancy, with a birth weight of 2430 g and a length of 49 cm. The parents noticed noisy breathing beginning in the first month of life. In addition to spinal deformities, the patient exhibited adenoidal hypertrophy and chronic rhinitis starting at 6 months of age.

On physical examination at 2 years and 10 months, his height was 86 cm (SDS: ⁣′1.84) and weight was 12.7 kg (SDS: −0.97). Clinical features included a short neck, kyphoscoliosis, hip dysplasia, flexion contractures of proximal interphalangeal joints of both hands, and contractures of the knee joints. The patient had coarse facial features with frontal bossing, a wide nose with a low nasal bridge, gingival hypertrophy, and deformation of cranial bones. Hepatomegaly was observed, with the liver palpable 3 cm below the costal margin, along with an umbilical hernia. The patient exhibited delayed speech, possessing a vocabulary of approximately 10 words, although understanding addressed speech at everyday level. No additional instrumental studies were carried out.

Biochemical analysis revealed ASB deficiency (0.01 *μ*mol/L/h) and slight increase of urinary dermatan sulfate (89.1 mg/mmol creatinine, normal range 3.3–40 mg/mmol creatinine).

**Figure 2:**
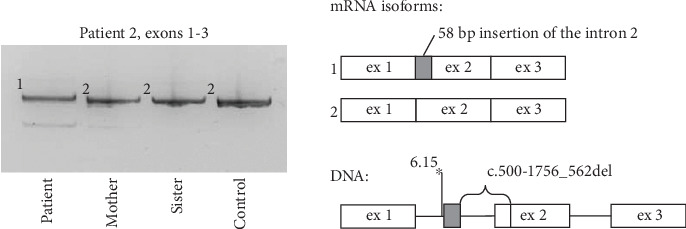
Molecular genetic findings in Patient 2. Visualization of the *ARSB* cDNA PCR products spanning Exons 1–3 by polyacrylamide gel electrophoresis and schematic representation of the studied locus at mRNA and DNA levels. Patient 2 is homozygous for the c.500-1756_562del, his mother is heterozygous, and his sister does not have this variant. The strength of the splice site (MaxEntScore) is indicated above the asterisk.

During Sanger sequencing of the patient's *ARSB* exons, an absence of amplification of the fragment spanning Exon 2 was detected. The subsequent sequencing of the *ARSB* cDNA fragment spanning Exons 1–3 revealed the chimeric isoform with deletion of 63 bp of Exon 2 and insertion of 58 bp of the Intron 1 (Figure 2). A deletion of the DNA fragment spanning this region was suspected and further detected by PCR and Sanger sequencing in the homozygous state in the patient and in the heterozygous state in patient's parents. The 1819 bp deletion (c.500-1756_562del), which removes the acceptor splice site of Exon 2, brings the strong intronic acceptor site closer to the rest of the exon and leads to the exonisation of the 58 bp fragment between them. At the RNA level, this event leads to the frameshift and premature stop-codon formation (corresponding to c.500_562delins58 (p.Gly167Alafs⁣^∗^2)).

### 2.3. Patient 3: Recurrent Deep Intronic Variant Leading to PE Activation

Patient 3 is a 10-year-old female born from consanguineous parents (first cousins). Her older sister presents with a similar clinical phenotype and was diagnosed during a family investigation. She was referred to a geneticist by an otolaryngologist due to frequent rhinitis and otitis media.

Patient was born following a second normal pregnancy, with a birth weight of 3800 g and a length of 51 cm. At 10 months of age, a physical examination revealed a Hurler-like phenotype characterized by coarse facial features, synophrys (medial eyebrow hypertrichosis), and short stature. She also exhibited flexion contractures of the interphalangeal, elbow, and knee joints. Umbilical and inguinal hernia were also seen. At 3 years, impairment of vision and hearing, noisy breathing, frequent respiratory infections, macroglossia, and hepatosplenomegaly were detected.

At 10 years old, a physical examination revealed significant growth retardation (height: 97 cm; SDS: −6.24) and failure to thrive (weight: 17 kg; SDS: −4.47). Notable findings included a pronounced chest deformity with an expanded lower thoracic aperture and claw-hand deformities. The patient presented Hurler-like facial characteristics, thick hair, macroglossia, difficulty with nasal breathing, and snoring during sleep. Abdominal examination revealed significant hepatosplenomegaly (liver palpable 5 cm below the costal margin; spleen palpable 4 cm below the costal margin) and an umbilical hernia. Ophthalmologic examination demonstrated impaired pupillary function, eye enlargement, corneal clouding, and epithelial edema, leading to a diagnosis of secondary glaucoma.

Biochemical analysis revealed ASB deficiency (0.01 *μ*mol/L/h) and increase of urinary dermatan sulfate (408 mg/mmol creatinine, normal range 3.3–40 mg/mmol creatinine).

**Figure 3:**
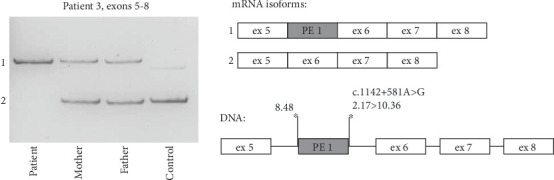
Molecular genetic findings in Patient 3. Visualization of the *ARSB* cDNA PCR products spanning Exons 5–8 by polyacrylamide gel electrophoresis and schematic representation of the studied locus at mRNA and DNA levels. Patient 3 is homozygous for the c.1142+581A>G PE-activating variant, and his parents are heterozygous. The strength of splice sites (MaxEntScore) is indicated above asterisks.

Sanger sequencing of the patient's *ARSB* gene failed to identify any causative genetic variants; therefore, the *ARSB* mRNA analysis was performed. RT-PCR of the cDNA fragment spanning Exons 5–8 revealed an additional high-molecular band, which turned out to be an insertion of a 125 bp fragment of the *ARSB* Intron 5 (chr5:78180827_78180951) (Figure 3). The fragment represents a PE (named PE 1) activated by the c.1142+581A>G variant, which was further identified in homozygous state in the patient and in heterozygous state in the patient's parents. This variant creates a strong donor splice site and leads to the insertion of the 125 bp PE 1 in a vast majority of the patient's mRNA molecules (corresponding to c.1142_1143ins125 (p.Ser381Argfs⁣^∗^3)).

### 2.4. Blocking of PEs Activated by the c.1142+581A>G and Polymorphic c.1142+671A>G Variants

Among the identified splice-altering variants, the c.1142+581A>G attracted our special attention because it is a recurrent mutation described earlier in two siblings with MPS VI [4]. In addition, the polymorphic c.1142+671A>G variant (with a total allele frequency of 24% in the Genome Aggregation Database (gnomAD)) located in the vicinity has been associated with altered *ARSB* gene splicing and even has been proposed to cause a subclinical phenotype [3, 10]. Such deep intronic PE-activating variants are certainly the relevant targets for genetic therapy based on antisense splice-modulating molecules (ASMs).

Of the various exon-skipping approaches, we chose those based on the expression of antisense ASMs as modU7snRNAs [7, 8] and as circRNAs [9]. These approaches are aimed at improving the stability and biodistribution of ASMs and allow an efficient delivery of ASMs to the wide range of tissues by adeno-associated viruses (AAV).

**Figure 4:**
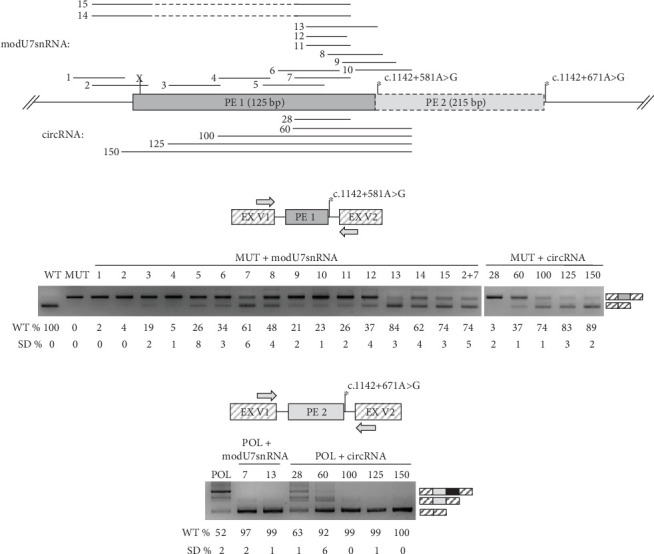
Testing of ASMs to block the identified PEs. (a) The scheme of the DNA locus encompassing two overlapping PEs with the location of antisense sequences incorporated into modU7snRNAs and circRNAs. PEs share the same acceptor but different donor splice sites strengthened by either the rare c.1142+581A>G variant or the polymorphic c.1142+671A>G variant. An insertion of both PEs into mRNA leads to premature stop-codon formation (indicated as “*X*”) and subsequent NMD. modU7snRNA 14 and 15 incorporate two consecutive antisense sequences with the difference that modU7snRNA 14 does not carry hnRNP A1 motifs at its 5⁣′ end. circRNAs are named corresponding to their antisense sequence length. (b, c) Results of minigene-specific splicing product analysis after cotransfection of minigenes and antisense vectors into HEK293T cells. RT-PCR was performed with minigene-specific primers located within constitutively spliced exons (EX V1 and EX V2) of the minigene vector. PCR products were visualized by 3% agarose gel electrophoresis and quantitatively analyzed by fragment analysis. “WT%” and “SD%” represent the mean amount and the standard deviation of the wild-type mRNA isoforms of three biological replicates. The c.1142+581A>G variant is indicated as “MUT” and the c.1142+671A>G variant as “POL”.

To test the efficacy of ASMs against identified PEs, we created minigenes, which reconstitute the observed splicing patterns of c.1142+581A>G and polymorphic c.1142+671A>G variant. The c.1142+671A>G variant creates the strong donor splice site and leads to the insertion of 215 bp PE (named PE 2) which partially overlaps with the PE 1 as they share the same acceptor site (Figure 4(a)).

Next, we incorporated various antisense sequences targeting PEs into modU7snRNA and circRNA cassettes cloned into plasmid vectors (Figure 4(a)). Minigenes and antisense vectors were cotransfected into HEK293T cells and 48 h later minigene-specific splicing products were measured by PCR and fragment analysis (Figures 4(b) and 4(c)).

Initial modU7snRNA screening against PE 1 (modU7snRNAs 1–10) identified the most sensitive region within its body, which overlaps with the modU7snRNA 7 site. Even a slight shift of antisense sequence to the left (modU7snRNAs 5 and 6) or to the right (modU7snRNAs 8, 9, 10) or its shortening (modU7snRNAs 11–12) greatly reduced the efficiency of the PE blocking. Elongation of antisense sequence towards the donor splice site (modU7snRNAs 13) slightly improved the efficiency. circRNAs spanning the modU7snRNA 7 site demonstrated the comparable efficiency starting from the antisense sequence length of 100 bp.

Activation of PE 2 by the polymorphic c.1142+671A>G variant led to the 48% reduction of WT mRNA isoforms in the minigene assay. modU7snRNAs 2, 7, and circRNAs with sequence length starting from 100 bp almost completely restored the WT splicing (Figure 4(c)).

## 3. Discussion

In this study, we described the application of RNA analysis and a number of molecular techniques for confirmation of diagnosis in three MPS VI patients and made the first steps towards personalized genetic therapy based on antisense ASMs.

The phenotypes of our patients represent the typical severe form of MPS VI in which the first symptoms appeared before 2 years of age. In addition to characteristic osteoarticular signs (dysostosis multiplex), coarsened facial features, hepatosplenomegaly, and eye disease, Patient 1 and 2 have reduced neurocognition, which is not common for MPS VI [1, 11]. The severe reduction of ASB activity and increase of urinary GAGs were detected in all three cases which agrees well with the type of identified mutations.

Patient 1 is a compound heterozygote for pathogenic nonsense variant c.966G>A (p.Trp322⁣^∗^) and insertion of the *LHFPL2* gene fragment in the *ARSB* intron 4, derived from NAHR.

Recent studies identified that 4.8%–9.5% of the human genome contain copy number variations, the significant part of which occur during NAHR events [12]. NAHR is a process of recombination between nonallelic sequences of DNA, mediated by the presence of low-copy repeats or segmental duplications with high sequence similarity (often more than 97%) [13]. Thousands of NAHR hotspots are distributed across the human genome and share some common features: presence of DNA regions, prone to double-strand breaks (palindromes, minisatellites and transposons) [14], enrichment of the histone methyl-transferase *PRDM9* binding sites [15] and partial proximity of recombination partners [16]. NAHR can cause gene deletions, duplications, inversions, or conversions and, if the dose-sensitive gene is affected, leads to genomic disorders [17].

The identified rearrangement involving the *ARSB* and the *LHFPL2* genes demonstrates the main hallmarks of NAHR, which are the presence of highly homologous L1 class transposons at recombination breakpoints and their close spatial location (Figures 1(d) and 1(e)). Considering the location of the *LHFPL2* fragment insertion deep in the intronic space, its pathogenicity was not obvious. Comprehensive RNA analysis started with the detection of characteristic allelic imbalance and expression pattern and ended with an identification of the exact 3⁣′ end of the chimeric mRNA isoform by RNA-seq and 3⁣′ RACE, established the molecular genetic mechanism of pathogenesis of the NAHR-derived insertion, which is PTT. PTT occurred at the polyA signal located at the 3⁣′ end of the MLT1A1 element—retrotransposon from the long terminal repeat (LTR) family. In the *LHFPL2* Intron 3, this element is located in the reverse orientation relative to the gene, but after insertion in the *ARSB* Intron 4, it was settled in the forward orientation on the sense strand and became able to terminate transcription by its strong polyA signal.

Transcription termination by transposons is a widespread process in the mammalian genomes with about 28% of human genes having at least one transposon-derived transcription termination signal, among which 70% lead to the synthesis of truncated transcripts [18]. Sense-oriented transposons can reduce genes' expression and are subjected to the negative evolutionary selection [19, 20]. PTT is also recently associated with a number of genetic diseases [21–23].

We demonstrated, that an expression of the patient's *ARSB* full-length mRNA isoform is reduced to about 1% (Figure 1(b)) and is represented mainly by the isoform produced from the allele with the nonsense variant (Figure 1(a)). An expression from the allele with NAHR-derived insertion is reduced by about three times and is represented mainly by the chimeric mRNA isoform containing *ARSB* Exons 1–4. This isoform leads to the synthesis of severely shortened nonfunctional ASB protein.

In the case of Patients 2 and 3, amplification of the *ARSB* cDNA immediately revealed the molecular defects, which are the deep intronic variant c.1142+581A>G leading to PE activation and the intragenic deletion altering the integrity and splicing of the *ARSB* Exon 2. RNA analysis of both variants demonstrated the premature stop-codon formation, and no residual amount of WT *ARSB* mRNA isoform.

Genetic variants, which alter genes' splicing, remain highly underrepresented types of disease-associated mutations. Some of them are located deep in the intron and cannot be detected by common methods like Sanger sequencing or whole exome sequencing, and some of them are misclassified, for example, as missense variants. Functional analysis of such variants not only establishes their molecular-genetic mechanism of pathogenesis but also lays the foundation for genetic therapy based on antisense ASMs.

Extensive experience has been accumulated in the use of ASMs in exon-skipping therapy, which is summarized in recent consensus guidelines [24]. These guidelines focus primarily on the design and use of chemically modified RNAs, but a growing body of research points towards the relevance of approaches based on expression of ASMs as modU7snRNAs [7, 8] and circRNAs [9]. The large number of successful clinical trials of drugs based on AAV also adds relevance to these approaches, because small modU7snRNAs and circRNAs cassettes can be easily incorporated into AAV vectors. AAV-based delivery of ASMs also eliminates such disadvantages of synthetic antisense oligonucleotides as low cellular uptake, low specificity towards various tissues, and low half-life time [25].

Thus, we decided to apply the modU7snRNAs and circRNAs to (1) block the recurrent PE 1 to establish an approach for the personalized genetic therapy for patients with the c.1142+581A>G variant and (2) block the polymorphic PE 2 to reduce the nonproductive splicing and increase gene expression, which could be beneficial for some MPS VI patients [26].

The first step in the development of ASM is to determine the efficiency of its antisense sequence for correcting a specific splicing defect. Cotransfection of the minigene, which reproduces the splicing pattern of the WT and the mutant allele and ASMs in the model cell line, serves as a powerful technique for this aim. Expressing minigenes with c.1142+581A>G and c.1142+671A>G variants in the HEK293T cell line, we successfully reproduced the reported splicing alterations, which are insertion of 125 bp PE 1 in all mRNA molecules and insertion of 215 bp PE 2 in 48% of mRNA molecules (Figures 4(b) and 4(c)). To restore normal splicing, we coexpressed modU7snRNAs and ribozyme-assisted circRNAs with minigenes. Initial modU7snRNA screening identified the most effective target within PE 1, which is the modU7snRNA 7 site. Surprisingly, the 25 bp modU7snRNAs targeting PE's splice sites or predicted SC35 splicing factor motifs demonstrated the least effectiveness. ModU7snRNA 7 site overlaps with a cluster of exonic splicing regulatory sequences high score motifs (Figure S5). The most effective modU7snRNA 13 also covers this cluster together with a high score SC35 motif and a part of the PE 1 donor splice site; however, its long antisense sequence of 36 bp questions its specificity. circRNAs with sequence length starting from 100 bp demonstrated the comparable efficiency, which is consistent with previous studies [9]. The significant advantages of circRNAs over modU7snRNAs are their longer half-life time due to an absence of exonuclease-sensitive free ends, and the capacity of bearing very long antisense sequences as they could inhibit the modU7snRNAs expression. On the other hand, the disadvantages of lengthening the antisense sequence in terms of specificity should be analyzed in further experiments on patient-derived cells.

The polymorphic PE 2 led to a significant decrease in WT mRNA isoform, which can actually have a modifying effect on the phenotype of patients or even healthy individuals, as was suggested earlier [10]. Thus, blocking such polymorphic PEs is a valuable approach to increase the productive splicing and corresponding gene expression, which could be beneficial for a number of patients [26–28]. The previously most efficient modU7snRNA 7 and 13, and circRNAs with sequence length starting from 60 bp almost completely (> 90% of WT mRNA isoform) blocked the PE 2, probably due to its relatively low inclusion rate (Figure 4(с)). The c.1142+671A>G variant also creates a strong donor splice site as c.1142+581A>G, but the resulting PE 2, while sharing the same acceptor splice site, is almost two times longer, which probably affects its splicing efficiency. Overall, the obtained data on the most effective antisense sequences for PE 1 and PE 2 can be further used to incorporate corresponding ASMs into AAV and move towards the clinical studies for MPS VI therapy.

Considering the splicing alteration in Patient 1, which is also PE inclusion leading to PTT, the same exon-skipping approach could be proposed. The similar molecular-genetic mechanism of pathogenesis was reported earlier in patient with Batten's disease [29]. Researchers identified the PE derived from mobile genetic element insertion and leading to PTT. Blocking this PE with chemically modified oligonucleotides increased the WT splicing and restored the function of the patient's fibroblasts, which lead to the stunning example of personalized therapy development—drug Milasen. However, our current efforts to block the identified PE in patient's fibroblasts with modU7snRNAs incorporated into lentiviral particles do not restore the amount of full-length mRNA isoform (data not shown). The most probable reason for it is that the *LHFPL2* insertion is nearly 52 kb in length and contains many other mobile genetic elements able to terminate the transcription.

Patient 2 has the intragenic deletion, which removes the acceptor splice site and a part of Exon 2. Such alteration is the relevant target for SMART (spliceosome-mediated mRNA transsplicing) or RESPLICE (RNA-guided trans-splicing with Cas editor) approaches based on transsplicing [30, 31].

Overall, we hope, the results of our study will aid researchers in the diagnosis of MPS VI and will serve as the first step in the development of additional therapeutic options for a part of MPS VI patients.

## 4. Materials and Methods

### 4.1. DNA Analysis

Genomic DNA was extracted from whole blood with ethylenediaminetetraacetic acid (EDTA) using a GeneJET Genomic DNA Purification Kit (Thermo Fisher Scientific, Waltham, Massachusetts, United States). Sanger sequencing was performed on ABI PRISM 3500xL Genetic Analyzer (Thermo Fisher Scientific, Waltham, Massachusetts, United States). WGS of the patient's DNA was performed with TruSeq DNA PCR-free sample preparation kit on NovaSeq 6000 (Illumina, San Diego, California, United States). Sequence reads were aligned to the human reference genome GRCh37.p13 (hg19) using Burrows–Wheeler Aligner (http://bio-bwa.sourceforge.net/). Structural variants were called Manta (https://github.com/Illumina/manta).

Variants were named according to the *ARSB* reference sequence NM_000046.5 and GRCh37.p13 (hg19) genome assembly. Identified variants were classified according to The American College of Medical Genetics and Genomics guidelines [32] and were deposited in the ClinVar database (https://www.ncbi.nlm.nih.gov/clinvar/).

### 4.2. RNA Analysis

Total RNA was isolated from whole blood cells using a Leukocyte RNA Purification Plus Kit (Norgene, Thorold, Ontario, Canada). The first strand of cDNA was synthesized using ImProm-II Reverse Transcriptase (Promega, Madison, Wisconsin, United States) and oligo(dT) primers. Overlapping fragments of the *ARSB* cDNA were amplified by PCR and Sanger sequenced. Real-time qPCR was performed on QuantStudio 5 thermocycler with SybrGreen chemistry.

RACE technique was performed after the double-stranded cDNA synthesis with Mint RACE cDNA amplification kit (Evrogen, Moscow, Russia). To establish the 3⁣′ end of the patient's *ARSB* mRNA, three rounds of subtractive hybridization were performed using the Step-Out PCR technique [33], the Mint RACE primer set (Evrogen, Moscow, Russia), and three gene-specific primers. Deep sequencing of RACE products was performed with AmpliSeq technology on Ion S5 (Thermo Fisher Scientific, Waltham, Massachusetts, United States).

High-throughput RNA-seq isolated from primary fibroblast cultures of the patient and healthy control was performed by the Beijing Genomic Institution (BGI, Shenzhen, China). cDNA libraries were prepared using MGIEasy RNA Directional Library Prep Set and sequenced on a DNBSEQ-G400 using a paired-end run (2 × 100 bases). A minimum of 60 million reads with Q30 > 96% were generated from each library. The raw sequencing data was processed with the custom pipeline based on open-source bioinformatics tools. Raw reads were mapped using STAR v2.7.8a [34]. Reads were aligned to the human reference genome GRCh37.p13 (hg19) and separately to the genomic region containing the entire *ARSB* gene with an insertion identified by the whole genome and Sanger sequencing with corresponding gene models. StringTie v.2.1.1 was used for transcript assembly and quantification [35]. Gene expression was calculated in transcripts per million (TPMs). Coverage and splice junctions were visualized using a Sashimi plot in the Integrative Genomics Viewer (IGV) desktop application [36].

### 4.3. Minigene Assay and Splicing Correction

Studied variants with the surrounding genomic locus were cloned into the intron of the pSpl3-Flu2-mTK vector (Figure S6). Minigene plasmids were transfected into HEK293T cell line (ATCC number: CRL-3216) at 80% confluency in 24-well plates using 2 mkl of TurboFect Transfection Reagent (Thermo Fisher Scientific, Waltham, Massachusetts, United States). After 48 h, the RNA was extracted and reverse transcribed. The plasmid-specific cDNA was amplified with primers located within constitutively spliced exons of the vector.

modU7snRNAs genes were cloned into pcDNA3.1 vector as described previously (Figure S7 and Table S2) [37, 38]. All of modU7snRNAs except modU7snRNAs 14 contain an additional tail with two hnRNP A1 exonic splicing silencer motifs, that has been shown to improve the inhibitory effect of modU7snRNAs on exon recognition [39]. Antisense sequences of various lengths (mainly 25–30 bp) were designed to keep the GC-content about 50% and avoid repetitions of more than four nucleotides and secondary structures.

For expression of circRNAs, we applied the Tornado system based on two self-cleaving Twister ribozymes, which flank the tested antisense sequence [40]. An empty circRNA cassette with restriction enzymes cloning site was constructed by overlap-extension PCR of several 100 bp commercially synthesized DNA fragments. The cloning site was further used for the insertion of antisense sequences, which were amplified from the minigene vector (Figure S8 and Table S3).

Splice site scores were calculated using MaxEntScan (http://hollywood.mit.edu/burgelab/maxent/Xmaxentscan_scoreseq.html). SC35 splicing factor motifs were predicted by ESEfinder 3.0 (https://esefinder.ahc.umn.edu/cgi-bin/tools/ESE3/esefinder.cgi). Total splicing regulatory motifs distribution was calculated by HExoSplice (http://bioinfo.univ-rouen.fr/HExoSplice_submit/index.php).

## 5. Preparation of Fibroblast Cultures

Primary fibroblast cultures were obtained from forearm skin biopsies and were grown up in the proliferative medium “Amniokar” (PanEco Ltd., Moscow, Russia). Subsequently, they were subcultured in ordinary growth medium Dulbecco's modified Eagle medium (DMEM), supplemented with 15% fetal bovine serum (PanEco Ltd., Moscow, Russia). The obtained cultures are available in the Common Use Center “Biobank” (Research Centre for Medical Genetics, Moscow, Russia).

### 5.1. Biochemical Analysis

An extracted enzyme product and an internal standard were separated on a Phenomenex Fusion-RP 50 × 2.1‐mm, 4‐*μ*m column (Phenomenex, Torrance, California, United States) with LC-30 Nexera System (Shimadzu Corporation, Kyoto, Japan) and detected on a tandem mass spectrometer QTrap 4500 (ABSciex, United States) equipped with a positive electrospray ionization. The data were analyzed using data analysis software Analyst 1.7.2 (ABSciex, United States).

Dried blood spots quality control samples, IS, and substrate of I2S, NAGLU, GALNS, ARSB, GUSB, and TPP1 were obtained from PerkinElmer (Waltham, Massachusetts, United States).

A single 3 mm dried blood spot punch was placed into each well of a 96-well assay plate, followed by the addition of 30 *μ*L of a reaction mixture containing seven substrates and internal standards. The plate was sealed and incubated at 37°C with shaking at 250 rpm for 18 h. After incubation, 100 *μ*L of a 1:1 methanol:ethyl acetate solution was introduced to quench the reaction, and the resulting mixture was transferred into a 96-well deep-well plate.

Subsequently, 400 *μ*L of ethyl acetate and 300 *μ*L of an aqueous 0.5 M NaCl solution were added. The contents were thoroughly mixed by repeated pipetting (10 times) and then centrifuged at 1500 × g for 6 min to achieve phase separation. From the upper solvent layer, 200 *μ*L was drawn off and transferred into a fresh multiwell plate. This fraction was evaporated to dryness under a gentle stream of nitrogen at room temperature.

To prepare the samples for analysis, the dried residue was reconstituted with 100 *μ*L of a 20:80 (v/v) water:acetonitrile solution containing 0.2% formic acid. Finally, the samples were subjected to liquid chromatography–mass spectrometry (LC-MS/MS) analysis.

## Data Availability

The data was submitted to the ClinVar database (https://www.ncbi.nlm.nih.gov/clinvar/). Accession numbers are as follows: VCV001137700.7 for c.783G>A (p.Lys261=), VCV002686031.1 for c.1142+581A>G, VCV002686032.1 for c.1142+671A>G, and VCV001343391.1 for the recombination. The next-generation sequencing data was processed using freely available code described in the Materials and Methods section. The custom web-based NGS-data-Genome interface was used to visualize and filter the resulting data, and its code is available from the corresponding author upon reasonable request.

## Supporting Information

Supporting Information Additional supporting information can be found online in the Supporting Information section. Note S1: Analysis of the rare synonymous c.783G>A (p.Lys261=) variant by minigene assay. Figure S1: Results of minigene assay for the c.783G>A (p.Lys261=) variant. Note S2: Analysis of the recombination breakpoints. Sequence of the recombination boundaries in the *LHFPL2* locus. Table S1: Expression level of *ARSB* and the housekeeping gene *B2M* in TPM. Figure S2: The IGV browser window demonstrating allelic imbalance for the c.783G>A variant on the RNA-seq data. Figure S3: Sashimi plot from RNA-seq data demonstrating heterozygous deletion of the Exon 3 in *LHFPL2*. Figure S4: Results of the chimeric mRNA isoform analysis by rapid amplification of cDNA ends (RACE) technique. Note S3: The fragment of the *LHFPL2* insertion, where the transcription termination occurred. Figure S5: Location of the predicted splicing regulatory motifs within PE 1 and PE 2. Figure S6: The scheme of the minigene expression vector with the *ARSB* intron insert as an example. Figure S7: The scheme of the modU7snRNA expression vector and sequence of the modU7snRNA gene. Table S2: The tested antisense sequences incorporated into modU7snRNA. Figure S8: The scheme of the circRNA expression vector and sequence of the circRNA cassette. Table S3: The tested antisense sequences incorporated into circRNAs.
