# Supplementary material for: Functional Analysis of Complex Structural and Splice-Altering Variants in the ARSB Gene Towards the Personalized Antisense-Based Therapy for Mucopolysaccharidosis Type VI Patients
Source: Hum Mutat. 2025 Jan 10;2025:2250030. doi: 10.1155/humu/2250030 (PMC12267954; doi:10.1155/humu/2250030)
Supplement: Supporting Information — Additional supporting information can be found online in the Supporting Information section. Note S1: Analysis of the rare synonymous c.783G>A (p.Lys261=) variant by minigene assay. Figure S1: Results of minigene assay for the c.783G>A (p.Lys261=) variant. Note S2: Analysis of the recombination breakpoints. Sequence of the recombination boundaries in the LHFPL2 locus. Table S1: Expression level of ARSB and the housekeeping gene B2M in TPM. Figure S2: The IGV browser window demonstrating allelic imbalance for the c.783G>A variant on the RNA-seq data. Figure S3: Sashimi plot from RNA-seq data demonstrating heterozygous deletion of the Exon 3 in LHFPL2. Figure S4: Results of the chimeric mRNA isoform analysis by rapid amplification of cDNA ends (RACE) technique. Note S3: The fragment of the LHFPL2 insertion, where the transcription termination occurred. Figure S5: Location of the predicted splicing regulatory motifs within PE 1 and PE 2. Figure S6: The scheme of the minigene expression vector with the ARSB intron insert as an example. Figure S7: The scheme of the modU7snRNA expression vector and sequence of the modU7snRNA gene. Table S2: The tested antisense sequences incorporated into modU7snRNA. Figure S8: The scheme of the circRNA expression vector and sequence of the circRNA cassette. Table S3: The tested antisense sequences incorporated into circRNAs. [file 2250030.f1.docx]

**Supplementary note 1.**

**Analysis of the rare synonymous c.783G>A (p.Lys261=) variant by minigene assay**

The c.783G>A (p.Lys261=) variant was not described before and is located in patient’s DNA in cis-position with the nonsense c.966G>A (p.Trp322*) variant. According to the HExoSplice, it leads to alteration of exonic splicing enhancer motifs. As expression of the patient’s *ARSB* mRNA is severely reduced, the results of the end-point PCR could not fully reflect all of isoforms, some of which may contain isoforms with the exon 4 skipping due to the c.783G>A variant.

To analyze the effect of c.783G>A on splicing in the absence of nonsense mediated decay, we created minigenes containing the wild-type *ARSB* exon 4, the exon with the c.783G>A variant and the exon with deletion of the predicted splicing enhancer motif c.783_785del.

After visualization and sequencing of splicing products, we did not detect any significant difference between the wild-type and the mutant minigenes (Supplementary Fig. S1). Thus, considering the synonymous nature of the variant, and presence of the pathogenic variant in-cis, c.783G>A was classified as “likely benign” according to the ACMG guidelines.

**Supplementary Figure S1. Results of minigene assay for the c.783G>A (p.Lys261=) variant.**

**
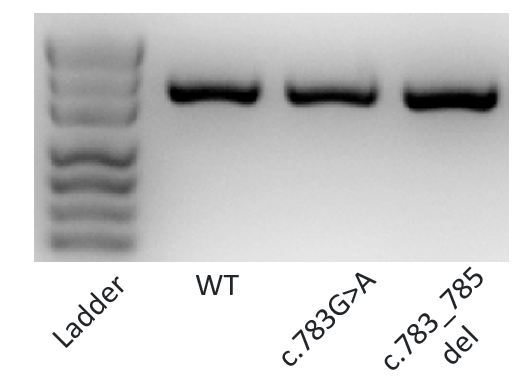
**

Visualization of the minigene-specific splicing products by 3% agarose gel electrophoresis.

**Supplementary note 2.** **Analysis of the recombination boundaries.**

**Sequences of the recombination boundaries in the *ARSB* locus:**

5` breakpoint (relative to the gene):

GTGCTCAGACAGAAGTGGAAGATGCTAGTTCTGGAAGACTGTATTAAATATCTCTAAGTGGCTCTTGTGATAACATTCCCATCAAGAATGCATGGGAGGCTGGGTCTGGTGGCTGACGCTTGTGATC_TCAAAAAGGCCCCAGTGGCTGAACGTGATCCCATGGAGAGTGGGGGGATCAGAAACAGCTAATGGGATGCAGAACTCATTTCGGGGCTGGGGAGGGGAGGATGCCGTACCAA

Sequencing primers:

ARSB_trans5_F: GCACCCACTGTCCACAATCC

ARSB_trans5_R: CGATTAGCATTGGTCTGAAGGTG

3` breakpoint (relative to the gene):

TTAGCATTGGTCTGAAGGTGGCCCAAGCCCTCTTTATCGTCCCTACCACACAGCCAGTGCAAATCCTTATCATCTCCCTTCTGGCTGTGGCCTCCCTGGCCCACCTTCAGAAAGAAGCCTTATGGCTCTGGAGGAAGCAATCT_GACAGATTTAAGAAATACATAGGAGGGGTGGAGCCAAGATGGCCGAATAGGAACAGCTCCAGTCTACAGCTTCCAACATGTGCGACGCAGAAGA

_ - recombination boundaries

Blue – *ARSB* sequence

Red – *LHFPL2* sequence

*ARSB*: chr5:78241273-78248429 - 7157 bp deletion and 51803 bp insertion of *LHFPL2*: chr5:77831940-77883742

Sequencing primers:

ARSB_trans3_F: GGCAACAGCCATTTACAGTGC

ARSB_trans3_R: GTGCTCAGACAGAAGTGGAAGA

**Sequence of the recombination boundaries in the *LHFPL2* locus:**

ACTCAAGTGAAAGAACTGGAAAAGTACCAATGCATCTATTTTCTTGGCCACTTGGAACCAGGGTACATCCTCATGTCTCCTGCGATTCTGGAACCACCGGAGGGAGGGGCAGAGAACATGAACTGGCCCTGG_TATGTTCCCAGGCTA_ACCATGTTTCCCAGGCTGGTGTTGAACTCCTGAACTCAAGTGACCTGCCTGCCTCGGCCTCCCAAAGTGCTGGGATCACAA_AGTTAGCTTTGGTATGGAAGCTGTGACTGGTGGTCTTCATAGAAGAAAGGCCAGGAATACCTCTGCACAGGTGTCCCACACGCCATGTGACTCCCATGTCCCCATGGAGAATGCCTAGGTCCCCGTAGGGCATAAACAGTTATTCTGATGCTCAGAACTCTGAGACTGGGGGGCTCCCACTTGTGATTTTGATACTAACCTATAGATTGTTATGAGTTCGTGTCAGA

_ - recombination boundaries

Blue – *ARSB* sequence

Red – *LHFPL2* sequence

Pink – non-specific sequence

*LHFPL2*: chr5:77831892-77883763 - 51872 bp deletion, insertion of unspecific 15 bp and 81 bp insertion of *ARSB*: chr5:78248357-78248437

Sequencing primers:

LHFPL2_trans_F: CGGACTCTGACACGAACTCAT

LHFPL2_trans_R: TGTTGCTTAAGGTCTCTGTCAG

**Table S1. Expression level of *ARSB* and the housekeeping gene *B2M* in TPM (transcripts per million).**

| Gene | Control | Patient |
| --- | --- | --- |
| *ARSB* | 21.5 | 4.6 |
| *B2M* | 1534.8 | 1855.1 |

**Supplementary Figure S2. The IGV browser window demonstrating allelic imbalance for the c.783G>A variant on the RNA-seq data.**


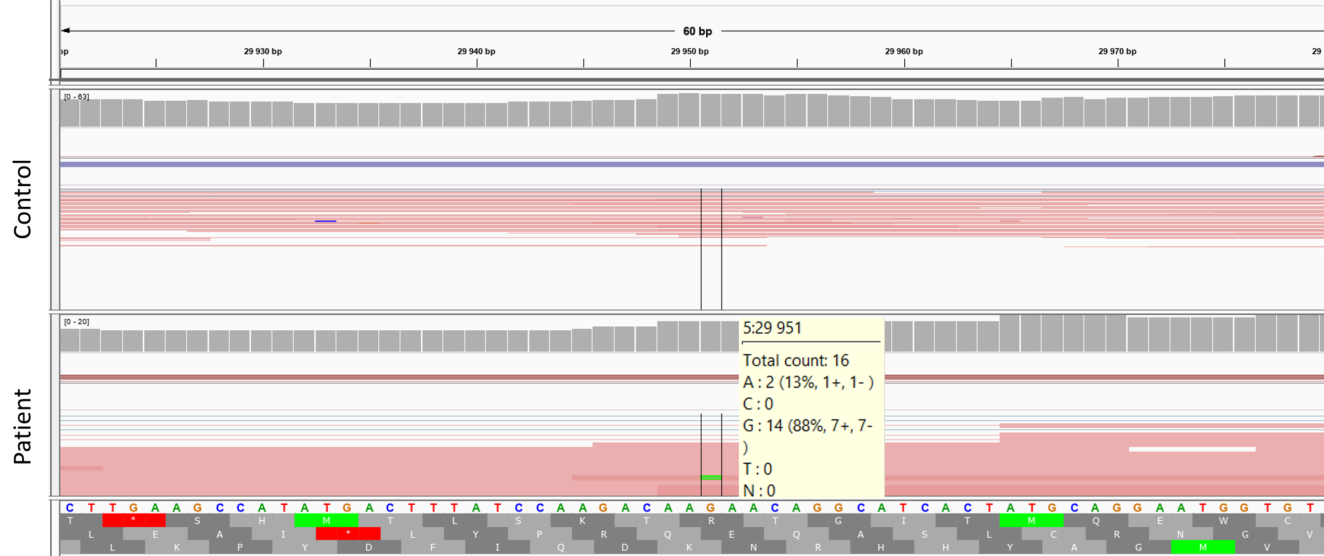


**Supplementary Figure S3. Sashimi plot from RNA-seq data demonstrating heterozygous deletion of the exon 3 in *LHFPL2*.**


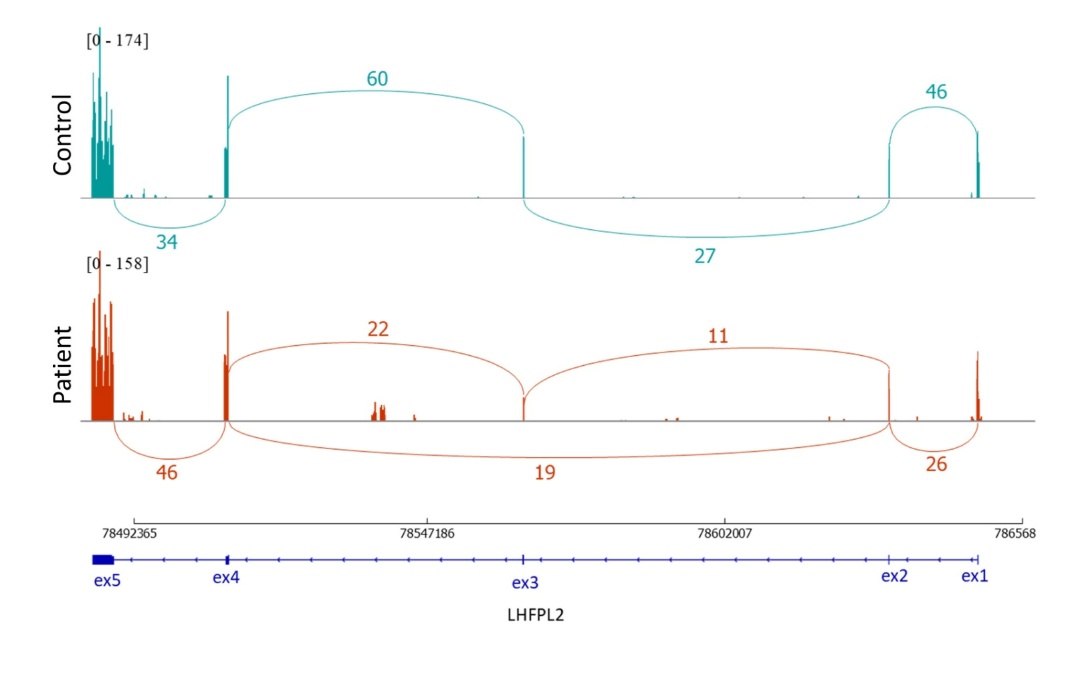


**Supplementary Figure S4. Results of the chimeric mRNA isoform analysis by rapid amplification of cDNA ends technique (RACE).**


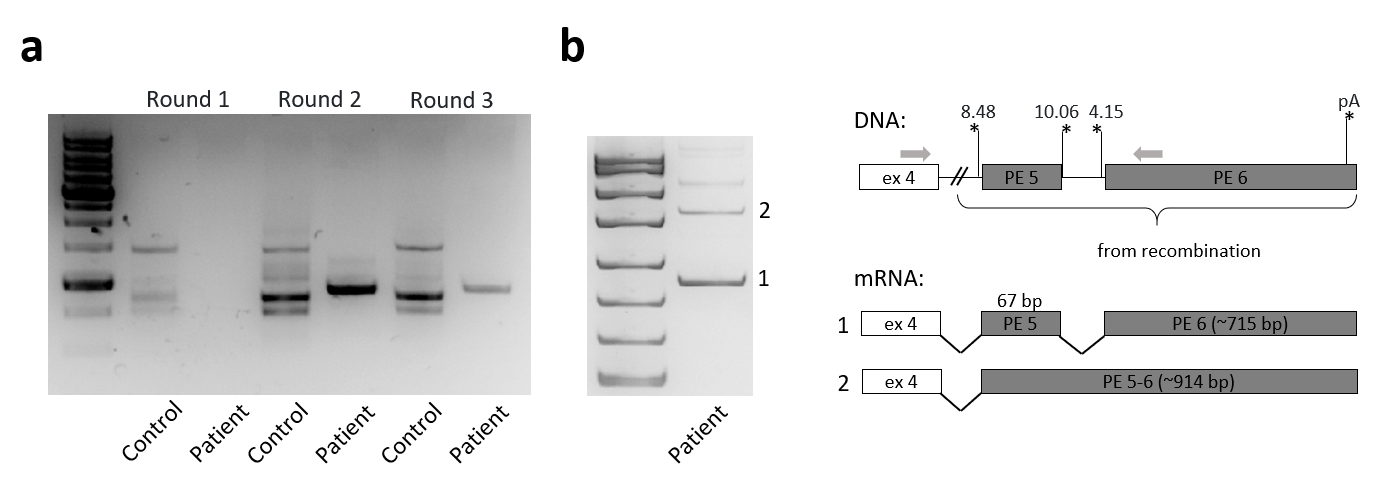


**a** Visualization of PCR products from three rounds of RACE reaction with forward primers located within exon 4 of *ARSB*. Unspecific products were detected in control sample probably due to long and complex wild type 3’ UTR and the chimeric mRNA isoform was detected in patient sample. **b** Visualization of the patient’s RACE product by polyacrylamide gel electrophoresis and a scheme of the mRNA structure. The strength of splice site (MaxEntScore) is indicated above asterisks. pA – polyadenylation signal.

**Supplementary note 3.**

**The fragment of the *LHFPL2* insertion, where the transcription termination occurred.** (corresponding to chr5:77834214-77835149, the orientation is relative to the gene)**:**

…..gccaggttctttgcttttct**ag**GGGGAAAAATCTCAGTGCATCTATCAATACTGGGCCCACATGTCAACGTGACAACAGGTGATTAGAG**gtaaga**agtggctgctctgttagacacaacacagtattctagtttgtcccagtctccaatacttcatattgcctcacaccatcctgtcttactcattcctgctatctacctgaatgtgtgactataccac**ag**GAGAGACTACAGTTGTGTCCAAAATCAACAACAACAACACCTGCCCATGACACCATCACCTGCATCTGCTTATTCTTTCCACTGACTCTGAAGGTGAGAAGCCACACCTGCACGGGCACAGTTTCACTTTCTCCATATGATCCCTGTGTGGGGACAGGAGCACCCACAGTATAAGCTGCCCCTTCATATAGTCCCATTGTCTTTGAGATGCACAGGTTGCCTCAGGTCTGCAGCCACACACCACGTATCCTTTGTACTGTCCAGTGACCTAGCACCAACCACACACCAGAAAGCATAATCCTTAAGAGCATGAGCCATAGTCTGCAACCTACTTCAAATTCCTAGCTCTGCTCTCCAATGGCGACATAATGACCCTAGGCAAGGCACTTGACACCTGCTATGGTTTGAATGATGATTCCCACCAAAATTCATGCTGAAATCCCCCACAAAATGGTATTAAGAGGTGTGGCCTTTGGGAGGTAATTAAGTCATGAGAGTCCCACCCTCACGGATGGGATCAGCATCCTTCCAAAAGGGCTTGGGGTTGAAGGGAGTGCCTGTGTACCCTTCTGCCCTCGGCCACGTGAAGACACAACAGCAAGGTGCCATCTTGGAAGCGGATAGCAGCCCTCACCCGACACCACTGCTGGCACCTTGGTCTTGGACCTCCCAGACTCCAGAACTGTGAGA**AATAAA**TGTCTGTTCTTTAAAATTAAAAAAAAAAAA….

The exonized sequences are in upper case. Splice sites and polyA signals are in bold.

**Supplementary Figure S5. Location of the predicted splicing regulatory motifs within PE 1 and PE 2.**

**
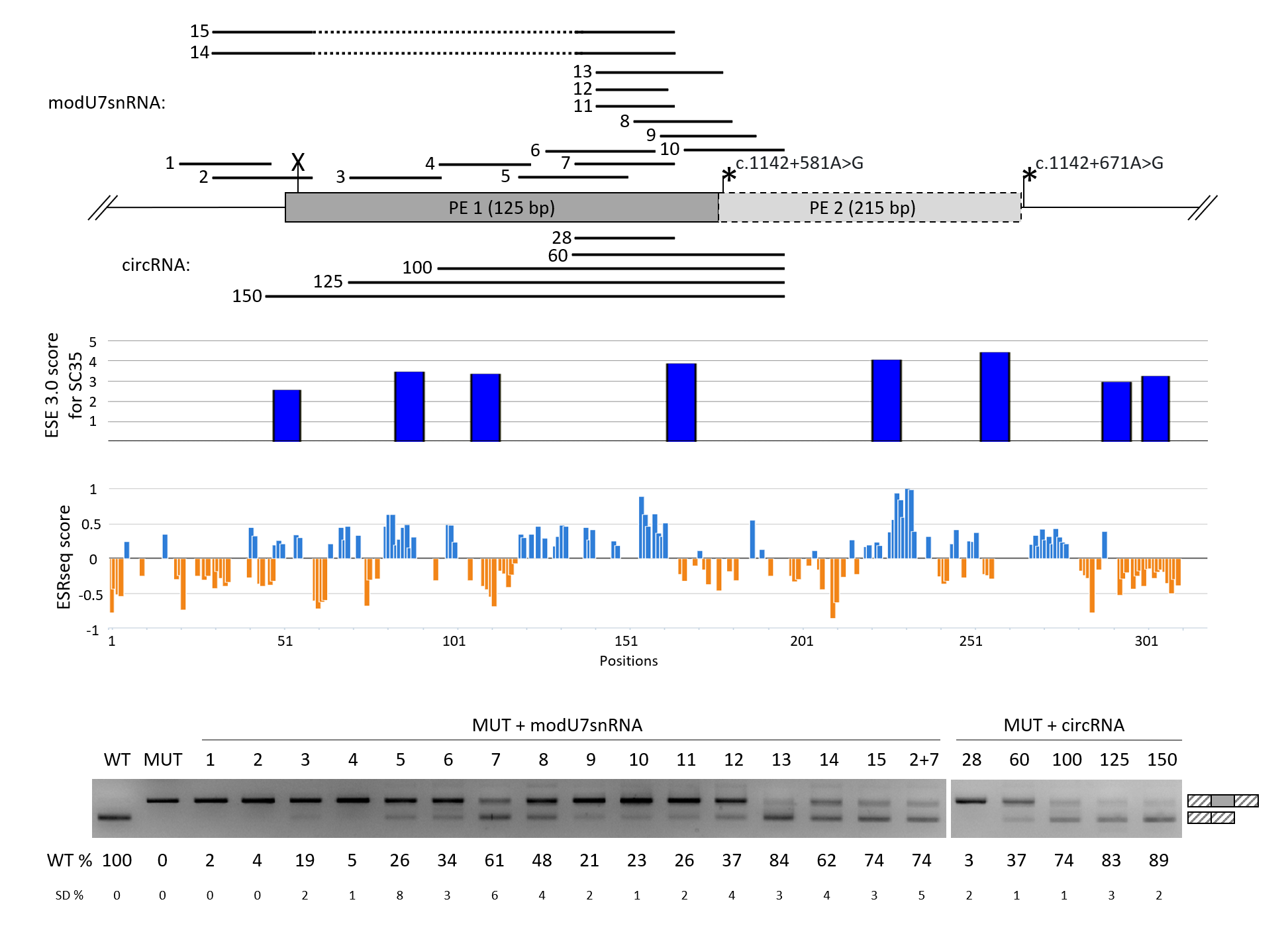
**

**Supplementary Figure S6. The scheme of minigene expression vector with the *ARSB* intron insert as an example.**

**Supplementary Figure S7. The scheme of modU7snRNA expression vector and sequence of modU7snRNA gene.**


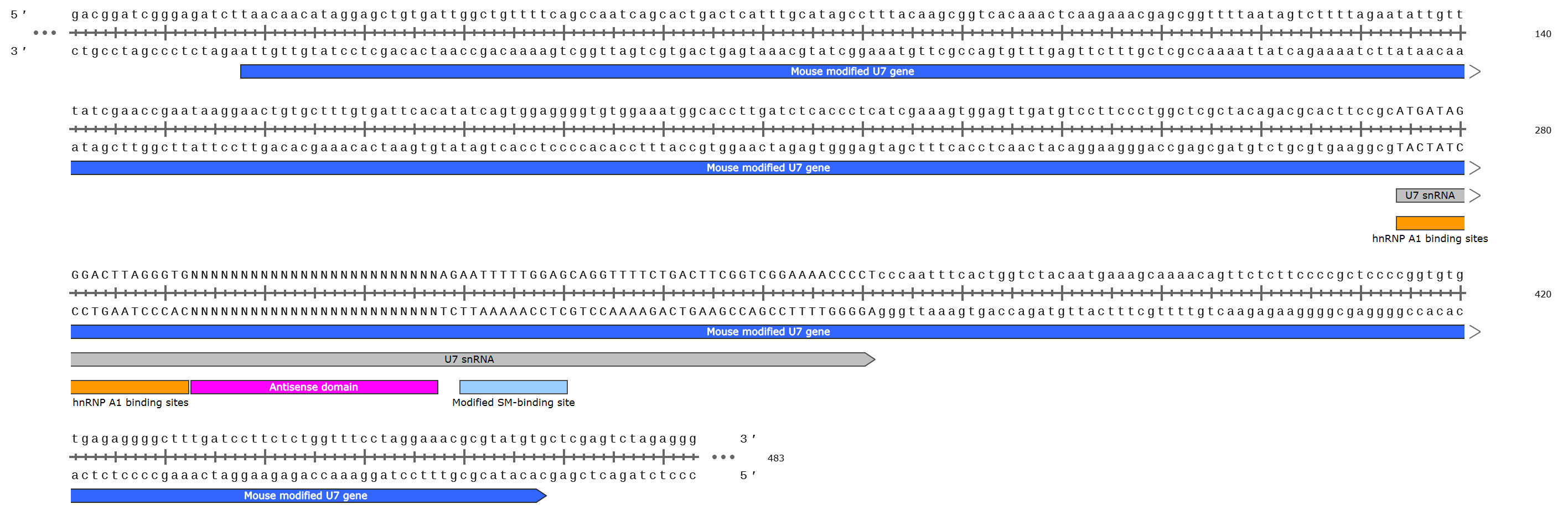


**Supplementary Table S2. The tested antisense sequences incorporated into modU7snRNA.**

| **N** | **SENSE** | **ANTISENSE** | **BP** |
| --- | --- | --- | --- |
| 1 | CCTACTATGTGCCATACCTTCTTCT | AGAAGAAGGTATGGCACATAGTAGG | 25 |
| 2 | GTGCCATACCTTCTTCTAGATGCTGAG | CTCAGCATCTAGAAGAAGGTATGGCAC | 27 |
| 3 | GAACAAGTCAGTCAAGATCCTGCCT | AGGCAGGATCTTGACTGACTTGTTC | 25 |
| 4 | CTCAGGAAGCTGTATTCTAGTTGGG | CCCAACTAGAATACAGCTTCCTGAG | 25 |
| 5 | GGGAGAAAGATGTTGGACAAATGAACACAC | GTGTGTTCATTTGTCCAACATCTTTCTCCC | 30 |
| 6 | GATGTTGGACAAATGAACACACAGATGAGC | GCTCATCTGTGTGTTCATTTGTCCAACATC | 30 |
| 7 | ACAAATGAACACACAGATGAGCAAGATG | CATCTTGCTCATCTGTGTGTTCATTTGT | 28 |
| 8 | AGCAAGATGACTGCCAGTTGTGGTA | TACCACAACTGGCAGTCATCTTGCT | 25 |
| 9 | GATGACTGCCAGTTGTGGTAAGTGC | GCACTTACCACAACTGGCAGTCATC | 25 |
| 10 | CAGTTGTGGTAAGTGCCAGGAAGGC | GCCTTCCTGGCACTTACCACAACTG | 25 |
| 11 | GAACACACAGATGAGCAAGATG | CATCTTGCTCATCTGTGTGTTC | 22 |
| 12 | GAACACACAGATGAGCAAG | CTTGCTCATCTGTGTGTTC | 19 |
| 13 | GAACACACAGATGAGCAAGATGACTGCCAGTTGTGG | CCACAACTGGCAGTCATCTTGCTCATCTGTGTGTTC | 36 |

**Supplementary Figure S8. The scheme of circRNA expression vector and sequence of circRNA cassette.**


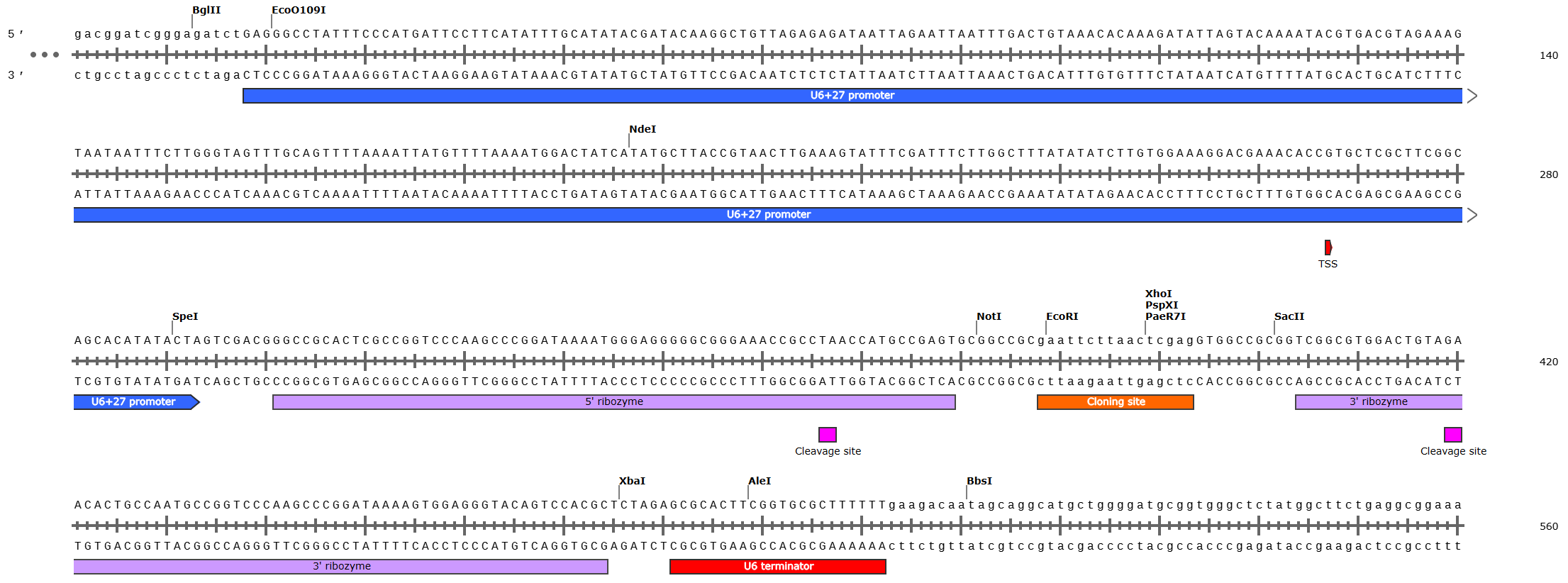


**Supplementary Table S3. The tested antisense sequences incorporated into circRNAs.**

| **BP** | **SENSE** | **ANTISENSE** |
| --- | --- | --- |
| 28 | ACAAATGAACACACAGATGAGCAAGATG | CATCTTGCTCATCTGTGTGTTCATTTGT |
| 60 | GACAAATGAACACACAGATGAGCAAGATGACTGCCAGTTGTGGTAAGTGCCAGGAAGGCAA | TTGCCTTCCTGGCACTTACCACAACTGGCAGTCATCTTGCTCATCTGTGTGTTCATTTGTC |
| 100 | CTCAGGAAGCTGTATTCTAGTTGGGGGAGAAAGATGTTGGACAAATGAACACACAGATGAGCAAGATGACTGCCAGTTGTGGTAAGTGCCAGGAAGGCAA | TTGCCTTCCTGGCACTTACCACAACTGGCAGTCATCTTGCTCATCTGTGTGTTCATTTGTCCAACATCTTTCTCCCCCAACTAGAATACAGCTTCCTGAG |
| 125 | GAACAAGTCAGTCAAGATCCTGCCTCTCAGGAAGCTGTATTCTAGTTGGGGGAGAAAGATGTTGGACAAATGAACACACAGATGAGCAAGATGACTGCCAGTTGTGGTAAGTGCCAGGAAGGCAA | TTGCCTTCCTGGCACTTACCACAACTGGCAGTCATCTTGCTCATCTGTGTGTTCATTTGTCCAACATCTTTCTCCCCCAACTAGAATACAGCTTCCTGAGAGGCAGGATCTTGACTGACTTGTTC |
| 150 | TTCTAGATGCTGAGAAATTAGGAATGAACAAGTCAGTCAAGATCCTGCCTCTCAGGAAGCTGTATTCTAGTTGGGGGAGAAAGATGTTGGACAAATGAACACACAGATGAGCAAGATGACTGCCAGTTGTGGTAAGTGCCAGGAAGGCAA | TTGCCTTCCTGGCACTTACCACAACTGGCAGTCATCTTGCTCATCTGTGTGTTCATTTGTCCAACATCTTTCTCCCCCAACTAGAATACAGCTTCCTGAGAGGCAGGATCTTGACTGACTTGTTCATTCCTAATTTCTCAGCATCTAGAA |
